# Acute generalized exanthematous pustulosis and Stevens-Johnson syndrome overlap due to hydroxychloroquine: a case report

**DOI:** 10.1186/s13256-020-02504-8

**Published:** 2020-11-03

**Authors:** Ivorie Coleman, Gabriel Ruiz, Sumir Brahmbhatt, Lindsay Ackerman

**Affiliations:** 1 Department of Medicine, University of Arizona College of Medicine, Phoenix, AZ USA; 2 Banner University Medical Center – Phoenix Division of Dermatology, University of Arizona College of Medicine, Phoenix, 475 North 5th Street, Phoenix, AZ 85004 USA

**Keywords:** Hydroxychloroquine, AGEP, COVID-19, Case report

## Abstract

**Background:**

Since the World Health Organization declared a global pandemic due to the novel coronavirus disease2019, there have been targeted efforts to establish management modalities. Hydroxychloroquine has been suggested as a possible treatment; however, it is associated with multiple adverse reactions. We report a rare case of a patient with acute generalized exanthematous pustulosis with Stevens-Johnson syndrome due to hydroxychloroquine. Acute generalized exanthematous pustulosis is characterized by acute onset of a generalized rash that is pustular and erosive in nature, affecting limbs; trunk; face; and, less often, mucosal membranes. Although rare, it is important to be mindful of this side effect because the diagnosis is often delayed, and the disease has the potential to be life-threatening.

**Case presentation:**

A 68-year-old American woman presented to our hospital with a painful, rapidly spreading rash. Its morphologic features included erythema multiforme–like lesions with extensive skin sloughing in various regions of the head, neck, and trunk and mucosal involvement. Her Nikolsky sign was negative, and she had no evidence of lesions on areas of skin trauma. Four weeks prior, she had been initiated on hydroxychloroquine for a presumed diagnosis of cutaneous sarcoidosis. Three punch biopsies of the head and neck area revealed subcorneal pustules consistent with acute generalized exanthematous pustulosis. Treatment began with high doses of methylprednisolone, leading to only minimal improvement of existing areas and ongoing spread to new areas. Treatment with intravenous immunoglobulin was initiated, at which point disease stability was achieved. The patient’s rash ultimately resolved, as did her cutaneous pain and pruritus.

**Conclusions:**

Among many potential adverse reactions involving hydroxychloroquine, cutaneous side effects are varied and can lead to significant morbidity or even death. The drug is currently being investigated in a multitude of trials for coronavirus disease2019 treatment, prevention, and prophylaxis after exposure to severe acute respiratory syndrome coronavirus 2. Acute generalized exanthematous pustulosis is a rare side effect of hydroxychloroquine, and even fewer cases demonstrate histologic evidence of acute generalized exanthematous pustulosis while clinically presenting with Stevens-Johnson syndrome. Patients who develop Stevens-Johnson syndrome/toxic epidermal necrolysis require best supportive care with aggressive fluid and electrolyte replacement and prevention of further breakdown of the skin barrier. With the potential of widespread hydroxychloroquine use, it is important that providers be aware of its potential severe adverse drug reactions.

## Introduction

Since the World Health Organization declared a global pandemic due to the coronavirus disease 2019 (COVID-19), there have been targeted efforts to establish management modalities. Aminoquinolines, such as chloroquine and hydroxychloroquine, have been suggested as possible agents [1]. These medications are traditionally used in the management of malaria and for dermatologic and rheumatologic conditions [2]. It is thought that aminoquinolines are positioned to treat viral infections due to mechanisms that mitigate viral activity [3]. Moreover, hydroxychloroquine has been suggested as a more appropriate choice than chloroquine [4]. Although the pharmacology may seem promising, this enticement should be coupled with caution because hydroxychloroquine is associated with multiple adverse reactions, including significant cardiotoxicity with prolongation of QTc and ventricular arrhythmias [5]. It is also known to cause vision-threatening toxic retinopathy [6].

We present a rare case of a patient with an adverse cutaneous reaction to hydroxychloroquine presenting with diffuse skin sloughing compatible with Stevens-Johnson syndrome (SJS). Histologic findings showed acute generalized exanthematous pustulosis (AGEP). Although rare, it is important to be mindful of the AGEP–SJS overlap presentation due to its often-delayed diagnosis and the potential to be life-threatening. AGEP is characterized by an acute onset of a generalized rash that is pustular and erosive in nature, affecting limbs; trunk; face; and less often, mucosal membranes. Noncutaneous features include fever, neutrophilia, and liver involvement with other features described in a few cases. The clinical course can vary [4] from self-limiting to requiring hospitalization. One prolonged case was reported that lasted up to 68 days [7]. In rare instances, AGEP pustules can convalesce, leading to bullae and skin sloughing akin to SJS.

## Case presentation

A 68-year-old American woman with a medical history significant for asthma, migraines, and proximal muscle weakness presented to our hospital as a direct admission for management of a painful rash that was rapidly spreading diffusely. Morphologic features included erythema multiforme–like lesions with extensive skin sloughing in various regions of the head, neck, trunk, and extremities. Her Nikolsky sign was negative, and she had no evidence of lesions occurring on areas of skin trauma. Her ophthalmologic examination showed conjunctival injection without photophobia. Her visual fields were intact. She reported no eye pain, blurry vision, or visual disturbances; thus, no slit-lamp examination was performed, and she had no indication of anterior uveitis or iridocyclitis. She had mucosal involvement with findings on the vulvovaginal mucosa, posterior oropharynx, and anal verge.

Four weeks prior to presentation, she had been started on hydroxychloroquine for a presumed diagnosis of cutaneous sarcoidosis. She was taking inhaled albuterol as needed and had no known drug allergies or other AGEP risk factors. She had a cutaneous lesion in the periocular area that was biopsied, revealing a diagnosis of granulomatous dermatitis, which was subsequently diagnosed as granulomatous rosacea.

**Fig. 1:**
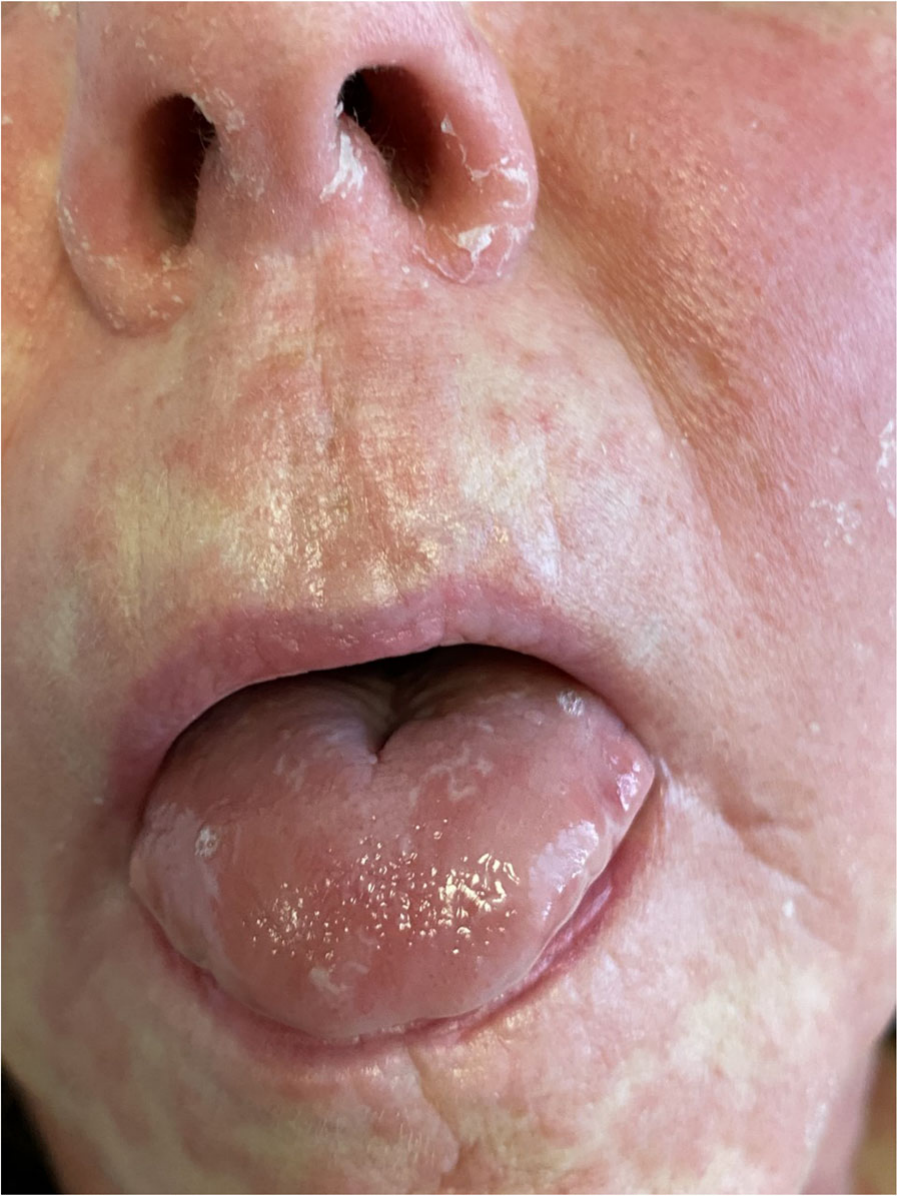
Characteristic mucosal involvement of acute generalized exanthematous pustulosis–Stevens-Johnson syndrome overlap with coalescing targetoid erythematous plaques visualized on surrounding face

**Fig. 2:**
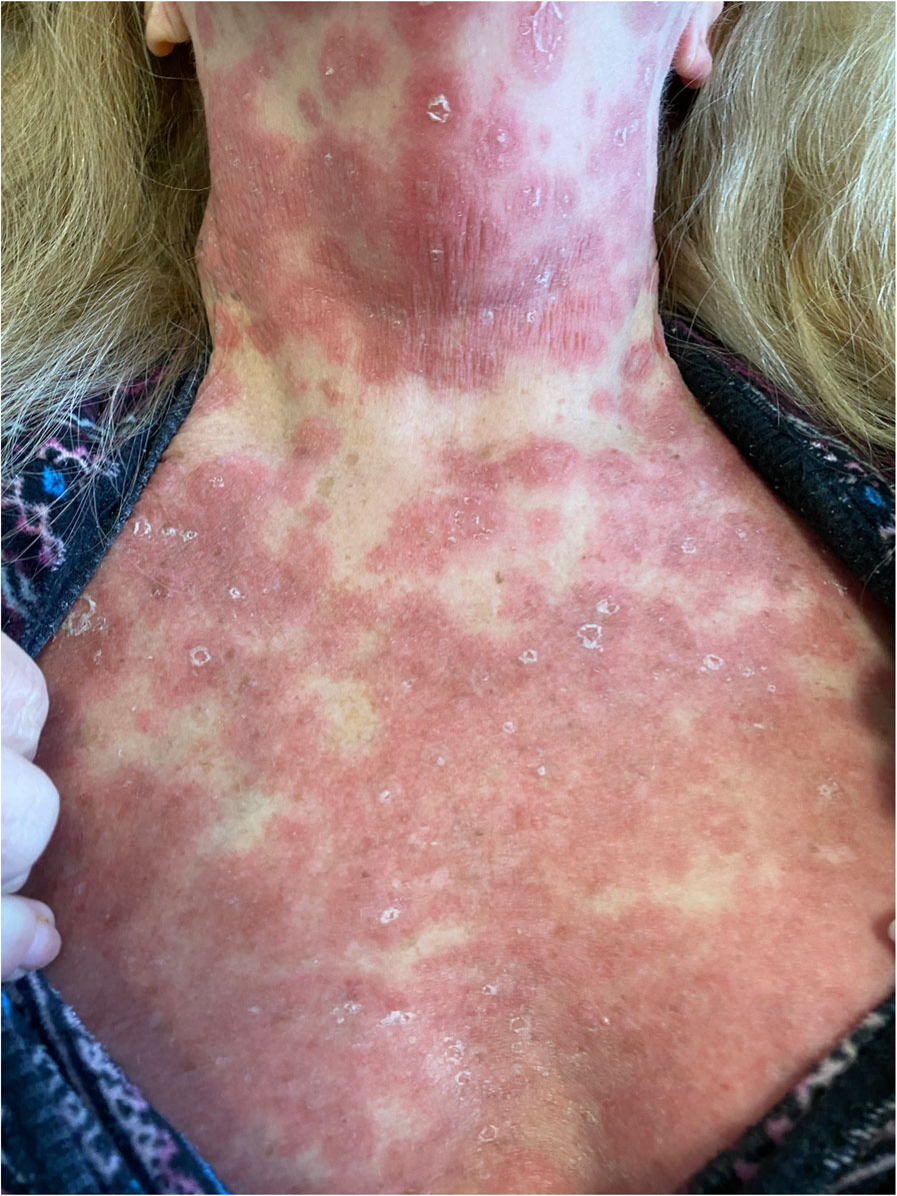
Coalescing targetoid erythematous plaques on chest and neck

Due to concern about her condition, three punch biopsies of the head and neck were performed. Biopsy results revealed subcorneal pustules consistent with AGEP (Figs. 1 and 2).

Treatment began with high doses of methylprednisolone twice daily over the course of 5 days, leading to only minimal improvement of existing areas and ongoing spread to new areas. Treatment with intravenous immunoglobulin was initiated at 400 mg/kg/day for 5 days, at which point disease stability was achieved, and she was safely transitioned to intravenous methylprednisolone once daily, which led to a prolonged taper with oral prednisone. The patient’s rash ultimately resolved, as did her cutaneous pain and pruritus.

Her hospital course was complicated by the direct impact of her cutaneous adverse event, including severe pruritus, generalized pain, extensive skin sloughing, dysuria, and painful defecation, as well as the indirect adverse complication of the reactivation of a latent varicella (zoster) infection. This was successfully managed with intravenous acyclovir. The patient was continuously concerned about permanent skin damage and recurrence despite discontinuing the offending agent. The management of pruritus and pain caused a great deal of anxiety and emotional distress. To better understand the skin involvement on a daily basis, the patient asked family and staff to capture photographs. The patient was later discharged to follow-up with the dermatology department as an outpatient.

## Discussion and conclusion

Among many potential adverse reactions involving hydroxychloroquine, cutaneous side effects are varied and can lead to significant morbidity or even death. The drug is currently being investigated in a multitude of trials for COVID-19 treatment [8], prevention [9], and prophylaxis after exposure to severe acute respiratory syndrome coronavirus 2 [10]. AGEP is a rare side effect of hydroxychloroquine, and even fewer cases demonstrate histologic evidence of AGEP while clinically presenting as SJS/toxic epidermal necrolysis (TEN). The typical presentation of AGEP is with a diffuse, “exanthematous” pustular rash. AGEP associated with SJS/TEN (of all causes) has a mortality similar to SJS [11], whereas more classic AGEP associated with hydroxychloroquine typically resolves quickly after stopping the medication [12].

Patients who develop SJS/TEN require best supportive care with aggressive fluid and electrolyte replacement and prevention of further breakdown of the skin barrier [13]. There is no known genetic predisposition for the development of AGEP, and the only identified risk factors are medication use or antecedent viral infection [14, 15].

We report a rare case of a patient with AGEP–SJS overlap that failed to respond to only supportive care, ultimately requiring both intravascular steroids and intravenous immunoglobulin. Herein lies the necessity for caution when hastily using hydroxychloroquine to manage the current global pandemic. The executive group of the World Health Organization Solidarity Trial recently decided to implement a temporary pause of the hydroxychloroquine arm within the trial as a precaution while the safety data of the drug are being reviewed [16]. This arm has since been resumed [17]. With the possibility of widespread hydroxychloroquine use, it is important that providers be aware of the possibility of severe adverse drug reactions to hydroxychloroquine, especially those who need aggressive intervention to avoid potentially irreversible consequences.

## Abbreviations

AGEP: Acute generalized exanthematous pustulosis
SJS: Stevens-Johnson syndrome
TEN: Toxic epidermal necrolysis
